# MicroRNA-98 inhibits the cell proliferation of human hypertrophic scar fibroblasts via targeting Col1A1

**DOI:** 10.1186/s40659-017-0127-6

**Published:** 2017-06-19

**Authors:** Sheng Bi, Linlin Chai, Xi Yuan, Chuan Cao, Shirong Li

**Affiliations:** Department of Plastic and Reconstructive Surgery, Southwest Hospital, Third Military Medical University, 29 Gaotanyan Main Street, Shapingba District, Chongqing, 400038 China

**Keywords:** miR-98, Col1A1, Apoptosis, Proliferation, HSFBs

## Abstract

**Background:**

Hypertrophic scarring (HS) is a severe disease, and results from unusual wound healing. Col1A1 could promote the hypertrophic scar formation, and the expression of Col1A1 in HS tissue was markedly higher than that in the normal. In present study, we aimed to identify miRNAs as post-transcriptional regulators of Col1A1 in HS.

**Methods:**

MicroRNA-98 was selected as the key miRNA comprised in HS. The mRNA levels of miR-98 in HS tissues and the matched normal skin tissues were determined by qRT-PCR. MTT and flow cytometry were used to determine the influence of miR-98 on cell proliferation and apoptosis of HSFBs, respectively. Col1A1 was found to be the target gene of miR-98 using luciferase reporter assay. Luciferase assay was performed to determine the relative luciferase activity in mimic NC, miR-98 mimic, inhibitor NC and miR-98 inhibitor with Col1A13′-UTR wt or Col1A13′-UTR mt reporter plasmids. The protein expression of Col1A1 in HSFBs after transfection with mimic NC, miR-98 mimic, inhibitor NC and miR-98 inhibitor were determined by western blotting.

**Results:**

The mRNA level of miR-98 in HS tissues was much higher than that in the control. Transfection of HSFBs with a miR-98 mimic reduced the cell viability of HSFBs and increased the apoptosis portion of HSFBs, while inhibition of miR-98 increased cell viability and decreased apoptosis portion of HSFBs. miR-98 inhibitor increased the relative luciferase activity significantly when cotransfected with the Col1A1-UTR reporter plasmid, while the mutant reporter plasmid abolished the miR-98 inhibitor-mediated increase in luciferase activity. Western blotting revealed that overexpression of miR-98 decreased the expression of Col1A1.

**Conclusions:**

Overexpression of miR-98 repressed the proliferation of HSFBs by targeting Col1A1.

## Background

Hypertrophic scarring (HS) is a serious disease, and results from unusual wound healing. It has excessive deposition of extracellular matrix [1]. Hypertrophic scar fibroblasts (HSFBs) often show vicious characteristics, such as excessive deposition and proliferation [2]. Basic fibroblast growth factor (bFGF) is reported to promote mitosis and to have an effect on endothelial cells [3]. FGF-2 could regulate myocardial infarct repair and could influence cell proliferation, scar contraction, and ventricular function [4]. When damage occurs, scar tissue forms [5]. Study showed bFGF could alleviate the scar of the rabbit ear model in wound healing [6]. Collagen type I (Col1) is the main structural element of the extracellular matrix (ECM). It served as a critical role in the development and progression of HS and the expression of Col-1 level was increased in HS tissues [7, 8]. Guofang et al. reported the production of Col1 was inhibited by miR-181c knockdown or miR-10a overexpression in HFs [9]. Xie et al. reported antisense oligodeoxynucleotide (ASODN) was effective in downregulating type I collagen gene expression and could prove to be useful in the treatment of scars [10]. Fibroblast collagen (Col) synthesis appears to be downregulated by keratinocyte-derived cytokines. Fibroblast growth factors and proinflammatory cytokines appear to be able partially to overcome this downregulation and to increase collagen synthesis [11].

MiRNAs have putative roles in the regulation of myofibroblasts and thus playing a part in hypertrophic scarring of the skin. Previous study showed miRNAs could putatively regulate proteins with a known role in myofibroblast regulation and function, for example, collagen type I (Col1A1) [12]. MicroRNAs (miRNAs) are single-stranded RNA molecules and can influence cell proliferation and differentiation [13]. They can bind to the 3′-UTR of cognate mRNAs, which can lead to the degradation of mRNA [14]. Many studies showed they had roles in a lot of diseases, such as skin inflammatory disorders [15, 16]. Recently, some miRNAs have been reported to participate in HS. MicroRNA 98 is reported to be related with many cancers. It can suppress tumor angiogenesis by influencing the level of matrix metalloproteinase-11 [17]. Its expression has the potential predictive value in formalin-fixed paraffin-embedded tissue from patients with breast cancer, and can be used as a diagnostic mark [18]. In mice models, there is a negative correlation between miR-98 and IGF-1 [19]. MiR-98 has been demonstrated to mediate the antihypertrophic effect of thioredoxin (Trx1) [20].

In present study, we aimed to explore the effect of miR-98 on proliferation and apoptosis of HSFBs and the molecular mechanism.

## Methods

### Tissue samples

Twenty HS tissue samples and matched normal skin tissues were obtained from twenty different patients in Hospital of Shanghai Jiaotong from May 2011 to June 2015. The written informed consents had been signed by all patients in advance. The experiments were approved by ethics committee of Central South University (the ethics certificate number is CSUEC 2011-094). We separated samples into three groups: sample 1 was stored in 4% paraformaldehyde solution; sample 2 was stored in liquid nitrogen and sample 3 was stored to isolate and cultivate fibroblasts.

### Cell culture

We obtained HSFBs and normal skin fibroblasts (NSFBs) (paired) from Ruijin Hospital, Affiliated to Shanghai Jiaotong University (Shanghai, China). We first cultivated fibroblasts in culture medium containing 0.5% fetal calf serum (FCS) after removing phenol red to train their adaptation in low serum concentration. Fibroblasts were cultivated in RPMI-1640 containing 10% inactivated FBS, penicillin (the concentration was 100 U/ml) and streptomycin (the concentration was 100 μg/ml) at 2D, 3D and Tis stage in a humidified cell incubator. The incubator contained 5% CO_2_ and the temperature in it was 37 °C.

### Identification of differentially expressed miRNA

Total RNAs were extracted from hypertrophic scar tissue and normal skin tissues using miRcute miRNA Isolation Kit (TIANGEN, China) according to the manufacturer’s instruction. The total 500 ng RNAs was subjected to an Agilent miRNA microarray analysis service (Bio Matrix Research, Nagareyama, Japan). Data analysis was done with the GenePix Pro software (LC Sciences). The miRNA array contained 2019 human probes. Probes with “present call” flag in at least one sample in both groups were used for further data analyses. Differences between groups were examined for statistical significance with unpaired Student *t* test. A *P* value <0.05 was considered statistically significant.

### Transfection of miR-98 mimic and inhibitor

The 2′-O-me-miR-98 mimic and 2′-O-me-miR-98 inhibitor were obtained from GenePharma (Shanghai, China). All the oligonucleotides were 2′-OMe modified. The transfection experiment was performed as previously described [21]. Briefly, cells were transfected with Lipofectamine 2000 (Invitrogen, CA, USA) and were analyzed 24 and 48 h after transfection.

### Quantitative real-time PCR analysis

RNA was obtained from HS tissue samples and matched normal skin tissues by mirVana miRNA isolation kit ThermoFisher Scientific (Austin, TX). Trizol was put into the kit and shaked well. The solution was transferred into 1.5 ml tubes using chloroform and centrifuged at 12,000×*g* for 15 min. Supernate was put into EP tubes again with isopropanol and centrifuged, and the precipitate was kept. Precipitate was treated with ethanol and DEPC was used to dissolve the precipitate. NanoDrop 1000 spectrophotometer (NanoDrop Technologies, Wilmington, Delaware, USA) was used to determine RNA concentration. The expression level was normalized using U6 small nuclear RNA by the 2^−ΔCt^ method. The ΔCt values were normalized to U6 level.

### Western blotting

Fifty micrograms of total protein extracts from HS cells transfected with miR-98 mimics or miR-98 inhibitor was loaded on SDS-PAGE gels for Western blotting. Western blotting was performed by a standard protocol. The mouse monoclonal anti-human Col1A1 antibody (R&D Systems Europe Ltd.) was diluted 1:500. Quantification of Western blot was performed by densitometry using the Storm 820 PhosphorImager.

### Luciferase assay

According to target prediction software microRNA.org to predict the binding site of miR-98. The fragment was inserted into the 3′-end of the firefly luciferase gene of the dual-luciferase miRNA target expression vector luciferase reporter vector (pGL3). The direct binding sites between miR-98 and Col1A1 3′ UTR were deleted by overlap extending PCR to construct pGL4.13-Col1A1-3′ UTR-mut.

### Cell counting kit-8 assay

Cell proliferation assay was performed according to the instruction of CCK-8 kit (Solarbio, Beijing, China). Cells at logarithmic phase were made into single-cell suspension and seeded to 96 well plate with 5 × 10^3^ cells. At 1, 2, 3, 4 and 5 days after seeding, 10 μl of CCK-8 solution mixed with 90 μl of DMEM was added into each well. After 2 h incubation, absorbance was measured at 450 nm.

### Flow cytometry

After transfection, cells were collected and made into single-cell suspension. The suspension was washed with PBS twice and fixed with 70% ethanol overnight. Propidium iodide (PI) single-stained reagent was added and placed avoiding light for 30 min. Flow cytometry (FCM) was used to determine the cell cycle in each group. The same method was used to collect cells, but fix was not performed with ethanol. AV/PI double-stained reagent was added and placed avoiding light for 10 min. FCM was used to determine apoptosis rate in each group. Each experiment was repeated for 3 times.

### Statistical analysis

Statistical evaluation for data analysis was determined by unpaired Student’s *t* test. Data were presented as the mean ± SD and *P* values <0.05 were considered significant.

## Results

### Hsa-miR-98 was down-regulated in hypertrophic scar

**Fig. 1:**
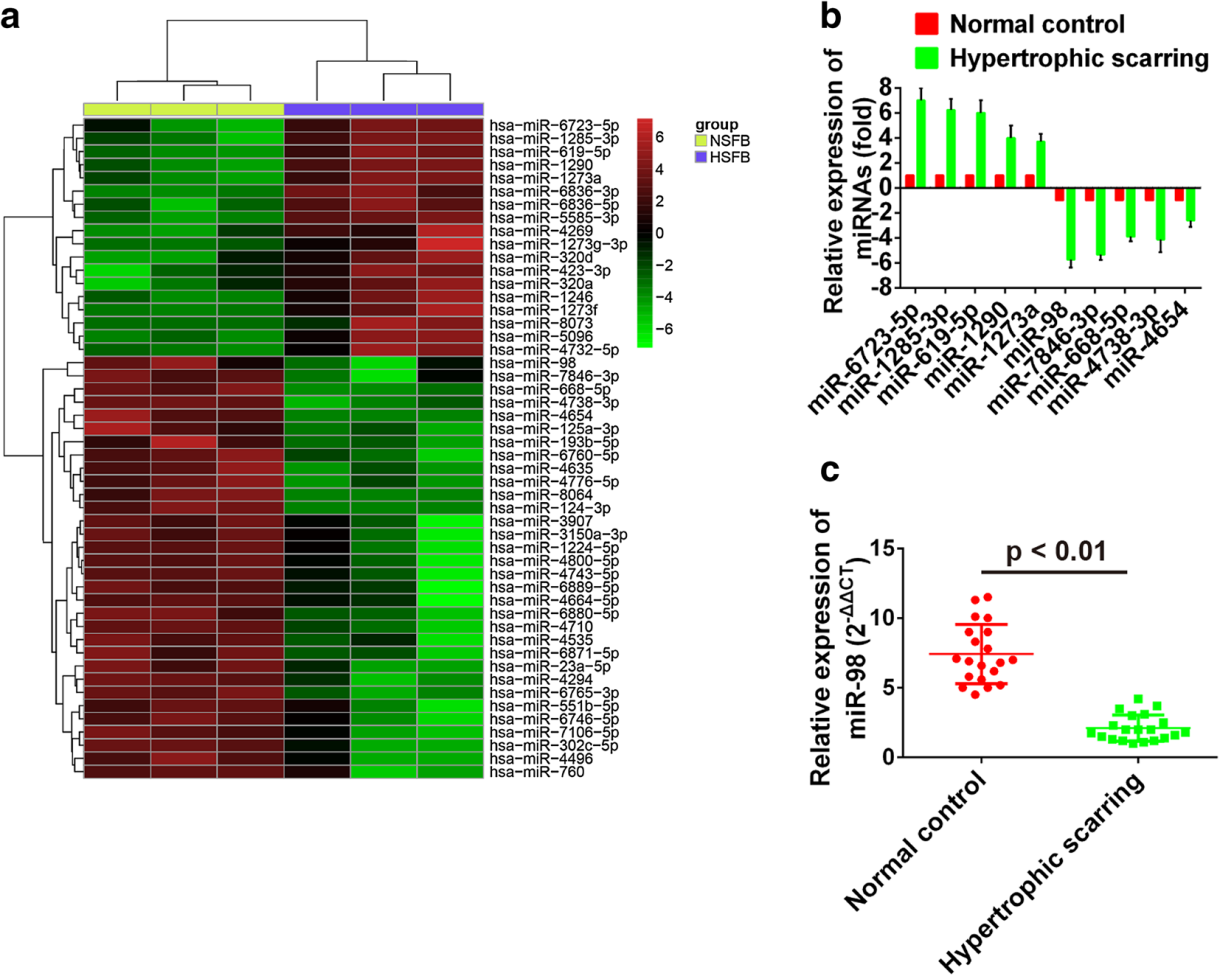
**a** Heatmap of a total of 50 miRNAs that were significantly down-regulated or up-regulated. For each miRNA the *red color* means an up-regulated expression of, and the *green color* means a down-regulated expression. **b** qRT-PCR was used to validate the results of microarray chips. **c** The relative expression of miR-98 in normal control and hypertrophic scarring tissues

To explore the key miRNAs consisted in HS, miRNAs data coming from human hypertrophic scars and mouse skin scar after wounding were downloaded from database and uploaded to GEO (http://www.ncbi.nlm.nih.gov/geo/query/acc.cgi?acc=GSE26213; http://www.ncbi.nlm.nih.gov/geo/query/acc.cgi?acc=GSE30913) to screen differential expressed genes. Results showed 18 miRNAs were up-regulated by more than 1.5-fold, and 32 miRNAs were down-regulated by more than 1.5-fold (Fig. 1a). To further validate the miRNA microarray results, 5 out of the most obvious up-regulated miRNAs (miR-6723-5p, miR-1285-3p, miR-619-5p, miR-1290 and miR-1273a) and 5 out of the most obvious down-regulated miRNAs (miR-98, miR-7846-3p, miR-668-5p, miR-4738-3p and miR-4654) were selected to determine their relative expression levels on hypertrophic scar tissues to verify the effect of chips. Results showed that relative expression values of miR-6723-5p, miR-1285-3p, miR-619-5p, miR-1290 and miR-1273a were above 0 and that relative levels of miR-98, miR-7846-3p, miR-668-5p, miR-4738-3p and miR-4654 were below 0 (Fig. 1b). This indicated the results of miRNA microarray chips from human hypertrophic scars were precise. Moreover, from the results, we obtained the most differentially expressed up-regulated miRNA was miR-6723-5p and the most differentially expressed down-regulated miRNA was miR-98. Previous study showed the expression of miR-98 in keloid fibroblasts was low. Therefore, miR-98 was selected for further study. Twenty hypertrophic scar tissues and the matched normal skin tissues were prepared and the relative expression of miR-98 in them was determined by qRT-PCR. Results showed the relative expression of miR-98 in hypertrophic scar tissues was significantly lower than that in the matched normal skin tissues (Fig. 1c).

### MiR-98 modulates hypertrophic scar fibroblast cell growth

We used qRT-PCR assay to determine the levels of miR-98 in NSFBs and HSFBs. The results were reported in Fig. 1c. As shown, hsa-miR-98 level of miR-98 in HSFBs was significantly lower than that in NSFBs (P < 0.001).

**Fig. 2:**
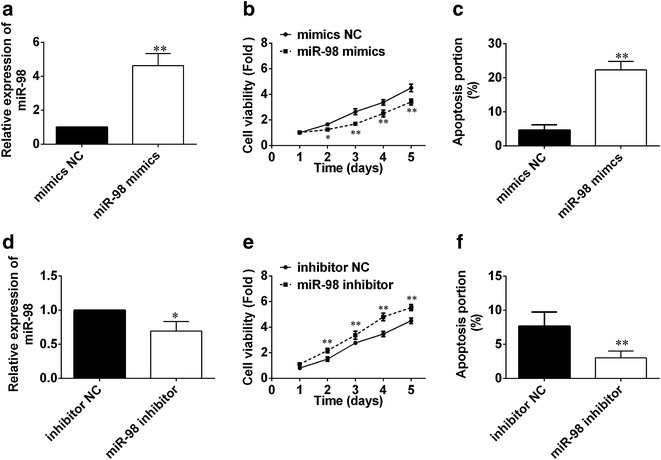
**a** The relative expression of miR-98 in HSFBs after transfection with mimics NC and miR-98 mimics. **b** Cell viability of HSFBs was determined after transfection with mimics NC miR-98 mimics for 1, 2, 3, 4 and 5 days. **c** The apoptosis portion of HSFBs after transfection with mimics NC miR-98 mimics. *P < 0.05 and **P < 0.01, compared with mimic NC group, the relative expression, cell viability or apoptosis portion of HSFBs in miR-98 mimics had significant difference. **d** The relative expression of miR-98 in HSFBs after transfection with inhibitor NC and miR-98 inhibitor. **e** Cell viability of HSFBs was determined after transfection with inhibitor NC and miR-98 inhibitor for 1, 2, 3, 4 and 5 days. **f** The apoptosis portion of HSFBs after transfection with inhibitor NC and miR-98 inhibitor. *P < 0.05 and **P < 0.01, compared with inhibitor NC group, the relative expression, cell viability or apoptosis portion of HSFBs in miR-98 inhibitor had significant difference

To explore the influence of miR-98 on HS, we transfected HSFBs with miR-98 mimic or inhibitor. After transfection for 24 h, we used qRT-PCR to determine the level of miR-98 in HSFBs. As shown in Fig. 2a, the level of miR-98 in miR-98 mimics group was significantly increased, while in miR-98 inhibitor group, the value decreased markedly (Fig. 2d). Then, we used MTT assay to determine cell viability. Results showed the cell viability of HSFBs reduced dramatically in miR-98 mimics (Fig. 2b), while cell viability in miR-98 inhibitor increased (Fig. 2e). FCM was used to determine the influence of miR-98 on cell apoptosis. Results showed apoptosis rate of HSBFs in miR-98 mimics group increased markedly compared with the control (Fig. 2c), while the apoptosis rate in miR-98 inhibitor decreased (Fig. 2f). All those indicated that miR-98 can influence the cell proliferation and apoptosis of HSFBs.

### The Col1A1 gene is a direct target of miR-98 in HSBFs

**Fig. 3:**
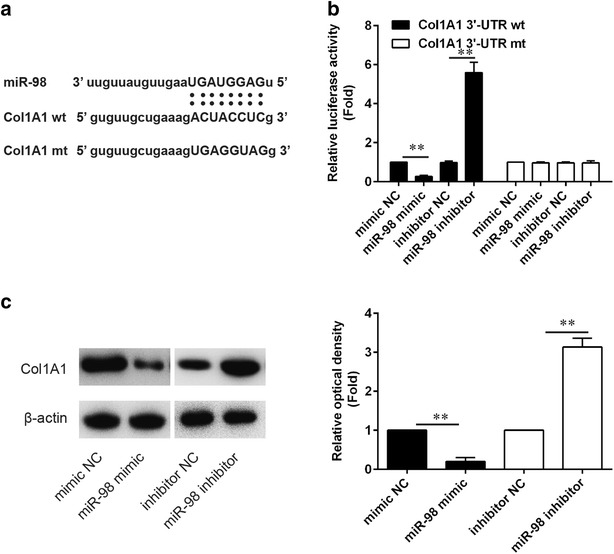
**a** Predicted miR-98 binding sites within the 3′-UTR of Col1A1mRNA. **b** The wt or mt reporter plasmid was cotransfected into HSBFs with miR-98 inhibitor, inhibitor NC, miR-98 mimic or mimic NC. Luciferase activity of pGL3-Col1A1 was increased significantly by miR-98 inhibitor and decreased markedly by miR-98 mimic. **c** Protein expression of Col1A1 after HSFB transfecting with miR-98 inhibitor, inhibitor NC, miR-98 mimic or mimic NC

Col1A1 was predicted to be the target gene of miR-98. In our study, results showed the wt of Col1A1 containing the potential miR-98 binding site (Fig. 3a). Luciferase assay was performed to determine the relative luciferase activity in mimic NC, miR-98 mimic, inhibitor NC and miR-98 inhibitor with Col1A13′-UTR wt or Col1A13′-UTR mt reporter plasmids. Results showed the relative luciferase activity in miR-98 mimic group with Col1A13′-UTR wt reporter plasmid was markedly lower than the mimic NC group with Col1A13′-UTR wt, while the relative luciferase activity in miR-98 mimic group with Col1A13′-UTR mt reporter plasmid had no significant difference with the mimic NC group with Col1A13′-UTR mt. Moreover, the relative luciferase activity in miR-98 inhibitor group with Col1A13′-UTR wt reporter plasmid was markedly higher than the inhibitor NC group with Col1A13′-UTR wt, while the relative luciferase activity in miR-98 inhibitor group with Col1A13′-UTR wt reporter plasmid had no significant difference with the inhibitor NC group with Col1A13′-UTR wt. Then, the protein expression of Col1A1 in HSFBs after transfection with mimic NC, miR-98 mimic, inhibitor NC and miR-98 inhibitor were determined by western blotting. Results showed there was a significant reduction in Col1A1 expression after transfection with miR-98 mimic and a markedly increase after transfection with miR-98 inhibitor (Fig. 3c), which kept pace with the results of luciferase reporter assay.

### MiR-98 was negative correlated with Col1A1

**Fig. 4:**
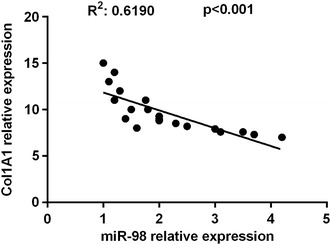
Inverse correlation of miR-98 and Col1A1 expression of in HS tissues

To further explore the regulatory roles of miR-98 in Col1A1 synthesis in vivo, we then determined the expression levels of miR-98 and Col1A1. An inversely relationship between miR-98 and Col1A1 was observed in skin scar (R^2^ = 0.6190, P < 0.001) (Fig. 4).

## Discussion

Hypertrophic scaring (HS) is a serious disease and can lead to grievous functional and esthetic defects [22]. The features of HSs include the proliferation of dermal tissue, of which there exists undue deposition of extracellular matrix proteins [23]. As reported, HS can result in substantial morbidity [24]. It plays a key role in preventing HS. Some research showed miRNAs play a key role in HS mechanism [25]. MiR-98 was reported to be an oncomir in recurrent nasopharyngeal carcinoma, ovarian cancer and prostate cancer [26]. However, studies on reporting the expression and functions of miR-98 in HS were few. In this study, results indicated that miR-98 regulated the apoptosis, cell viability of HS cells by targeting Col1A1. These findings help us to explore the mechanism underlying HS formation and therapeutic strategies for this disorder.

Collagen is extracellular matrix (ECM) component and its disorganized accumulation can result in scar formation [27]. The change of collagen was reported to have a key role in HS. The deposition of I and III collagen can result in HS [28]. The expression of Col1A1 in HS tissue was markedly higher than that in the control [29]. Col1A1 could promote the hypertrophic scar formation [30]. Some studies showed when ECM proteins (such as pro-Col1A1) increased, the excessive scar fibrosis occurred [31]. Scars are characterized by excessive collagen deposition, particularly types I and III collagen [32].

In our study, results showed miR-98 can regulate Col1A1 expression by targeting the 3′-UTR of Col1A1. After HSFBs transfected with miR-98 mimic, the expression of Col1A1 reduced, while the expression increased after transfection with miR-98 inhibitor. Moreover, after transfection with miR-98 mimic, the cell viability of HSFBs decreased and the apoptosis portion of HSFBs increased, while inhibition of miR-98 increased cell viability and decreased apoptosis portion of HSFBs. Fibroblast apoptosis had a key role in normal and pathological scar formation and the putative apoptosis-inducing factor curcumin affected fibroblast apoptosis and may function as a novel therapeutic [33]. It was known that 10-hydroxycamptothecin (HCPT) can prevent fibroblast proliferation, which further affect epidural scar adhesion after laminectomy in rats [34]. The presence or absence of scar was matched with the type of fibroblast generating the fetal wound matrix in a postnatal environment [35]. This suggested that HSFB proliferation was adjusted by decreased Col1A1 expression, which was resulted from the overexpression of miR-98. Those indicated that Col1A1 was a key downstream mediator of miR-98 in HSFBs.

In conclusion, our findings revealed that overexpression of miR-98 repressed the proliferation of HSFBs by targeting Col1A1. Enforced overexpression of miR-98 led to marked reduction of Col1A1 production, which indicated miR-98 was a new method to prevent scar. All those results indicate that miRNAs have a key role in skin fibroblasts.

## Abbreviations

HS: hypertrophic scarring
HSFBs: hypertrophic scar fibroblasts
bFGF: basic fibroblast growth factor
miRNAs: microRNAs
Trx1: thioredoxin
NSFBs: normal skin fibroblasts
FCS: fetal calf serum
RIPA: radio immunoprecipitation assay
BSA: bovine serum albumin
FCM: flow cytometry
ECM: extracellular matrix
HCPT: hydroxycamptothecin
